# Action potential evoked transmitter release in central synapses: insights from the developing calyx of Held

**DOI:** 10.1186/1756-6606-2-36

**Published:** 2009-11-25

**Authors:** Lu-Yang Wang, Michael J Fedchyshyn, Yi-Mei Yang

**Affiliations:** 1 Program for Neurosciences and Mental Health, The Hospital for Sick Children and Department of Physiology, University of Toronto, 555 University Avenue, Toronto, Ontario, M5G, 1X8, Canada

## Abstract

Chemical synapses are the fundamental units that mediate communication between neurons in the mammalian brain. In contrast to the enormous progress made in mapping out postsynaptic contributions of receptors, scaffolding structures and receptor trafficking to synaptic transmission and plasticity, the small size of nerve terminals has largely precluded direct analyses of presynaptic modulation of excitability and transmitter release in central synapses. Recent studies performed in accessible synapses such as the calyx of Held, a giant axosomatic synapse in the sound localization circuit of the auditory brainstem, have provided tremendous insights into how central synapses regulate the dynamic gain range of synaptic transmission. This review will highlight experimental evidence that resolves several long-standing issues with respect to intricate interplays between the waveform of action potentials, Ca^2+ ^currents and transmitter release and further conceptualize their relationships in a physiological context with theoretical models of the spatial organization of voltage-gated Ca^2+ ^channels and synaptic vesicles at release sites.

## Background

Information transfer between nerve cells is largely mediated by fast chemical neurotransmission across their junctions or synapses. Electric pulses or action potentials (APs or spikes) are initiated near the soma of presynaptic neurons and propagated along the axon to the nerve terminal where voltage-gated Ca^2+ ^channels (VGCCs) open and transiently raise intracellular Ca^2+ ^concentration. Binding of Ca^2+ ^to the release sensor (i.e. synaptotagmin) triggers membrane fusion between synaptic vesicles (SVs) and presynaptic active zones (AZs) via engagement of SNAREs. Upon opening of the fusion pore, neurotransmitters are unloaded into the synaptic cleft and activate postsynaptic receptors to generate excitatory or inhibitory postsynaptic potentials (EPSPs or IPSPs), ultimately affecting postsynaptic spike initiation and firing patterns that encode information.

Although it is well accepted that neurotransmission across chemical synapses is a highly conserved process in all species, our understanding of how the waveform of APs modulates Ca^2+ ^influx and quantal output is primarily based on classical work in invertebrate synapses from squid, crayfish and *Aplysia *[1-6]. This is mainly because typical mammalian central nerve terminals are too small to be readily accessible to electrophysiological analyses, limiting our ability to probe the intricate interplay between spike waveform, Ca^2+ ^currents, and transmitter release. Recently, optical recording techniques with fast Ca^2+^- and voltage-sensitive dyes, and new preparations that are conducive to direct electrophysiological recordings from nerve terminals have been developed, to study these variables and their crosstalk in several central synapses including the cerebellar granule cell-Purkinje cell synapses [7], the mossy fiber-CA3 synapses in hippocampus [8] and the calyx of Held synapses in the auditory brainstem [9-11]. These studies have provided the first quantitative description of the biophysical behavior of VGCCs in response to an AP, and the downstream coupling of Ca^2+ ^influx to vesicular release in mammalian central synapses. Since APs and synaptic transmission have been elegantly reviewed [12,13], this article will instead focus on a number of controversial issues with respect to the effects of AP on Ca^2+ ^channels and transmitter release.

First, in contrast to studies performed in invertebrate synapses where only a small fraction of VGCCs (~10%) are activated by an AP [2,3], work in mammalian central synapses demonstrates that an AP can effectively open a majority of presynaptic VGCCs (>70%) [8,14-17]. However, the mechanisms underlying this difference are not well understood. Second, the waveform of an AP is known to be altered by neuromodulators or to undergo activity- and development-dependent changes [6,18-20], but opposing views remain with respect to how AP width affects presynaptic VGCCs. In squid giant synapse, AP broadening appears to increase the number of activated VGCCs, but it alters mainly the gating kinetics of VGCCs in central terminals [21,22]. Third, in non-mammalian synapses, release of single vesicles can be triggered by the opening of as few as one channel [3,23-25] and AP broadening leads to linear increase in quantal output [3]. In contrast, at mammalian nerve terminals, the relationship between Ca^2+ ^influx and quantal output in response to AP broadening usually follows a highly nonlinear power function [8,14,15], implying that the cooperative action of multiple channels lead to the fusion of single synaptic vesicles [21,22,26,27]. These studies raise the possibility that different synapses may exhibit distinct coupling modalities between VGCCs and SVs, underpinning the heterogeneity of release probability (*Pr*). Finally, the peak Ca^2+ ^concentration experienced by Ca^2+ ^sensors on SVs in the vicinity of AZs was estimated to be more than ~100 μM in non-mammalian synapses but as low as ~10 μM in mammalian synapses during an AP [28-33]. Such a discrepancy in spatiotemporal profile of Ca^2+ ^transients is not fully understood.

## The giant calyx of Held synapse-a powerful experimental model

Recent studies using the calyx of Held synapse as an experimental platform have made tremendous progress towards addressing the aforementioned issues. This synapse at maturity faithfully relays inputs from the contralateral cochlear nucleus (CN) to the ipsilateral medial and lateral superior olives, where neurons encode the relative interaural timing/intensity differences of sound stimuli received at each cochlea as cues for sound localization [34]. From synapse formation at postnatal day 1-3 (P1-3) to the onset of hearing at P10-12 and final maturation around P16-18, this axosomatic synapse achieves the capacity of high-fidelity transmission at extraordinarily rates (up to 600 Hz) [20,34]. Its clearly defined "critical period of development" and rapid functional maturation make this synapse an ideal model for studying synaptic transmission and developmental plasticity. Meanwhile, as the waveform of presynaptic APs shortens dramatically within this developmental window, questions related to interplay between APs, Ca^2+ ^currents (I_Ca_) and transmitter release become relevant to physiological functionality of this synapse. The large dimension and compact structure of this synapse obviously present technical advantages for direct biophysical analysis of synaptic properties [9-11,26,35]. For example, since each postsynaptic neuron receives a single calyx input at the soma, where adequate voltage-clamp of pre- and postsynaptic elements can be simultaneously attained, the shape and size of the recorded currents can be reliably used for deconvolution analysis of the amount of transmitter release and the kinetics and density of postsynaptic receptors with minimal concern for voltage-clamp errors. The accessibility of both pre- and postsynaptic elements to patch electrodes offers the option of using either cell-attached non-invasive recording mode with excellent signal-to-noise ratio (seals at ~GΩ level) [36] or invasive whole-cell recordings. The measurements of current, voltage, and capacitance in designated intracellular ionic and buffer conditions facilitate direct studies of pre- and postsynaptic mechanisms [37]. In particular, simultaneous Ca^2+ ^imaging and photolysis of caged Ca^2+ ^makes quantitative cross-correlation analyses between Ca^2+ ^and quantal output possible, independent of Ca^2+ ^entry from the extracellular space [31,32]. Whole-cell capacitance recordings from the calyx provide means to directly study exocytosis and endocytosis independent of postsynaptic receptors while cell-attached capacitance recordings from the release face of the calyx make it possible to examine kinetics and conductance of fusion pore and VGCCs at release sites [38-40]. More importantly, presynaptic APs at the developing calyx of Held synapse undergo dramatic shortening in half-width, the quantal output (*Q*) is paradoxically maintained (in rats) or even markedly increases (in mice) [18,19]. Knowing that the input-output relationship at any given synapse can be described by a power function *Q ∝ [Ca*^2+^*]*^*n*^, where *n *denotes Ca^2+ ^cooperativity (3~5) [41], one would predict that spike narrowing should yield a much smaller Ca^2+ ^influx and therefore a nonlinear decline in *Q*. Contrary to this prediction, compelling experimental evidence demonstrated that the calyx of Held synapse displays impressive developmental adaptations, which compensate for the reduction in Ca^2+ ^and boost quantal output, in order to establish the robust high-fidelity character observed at this synapse [19]. Hence, investigation of mechanisms underlying such a compensation may not only unravel important answers for the physiological paradox at the developing calyx of Held synapse, but also insightful inferences for resolving long-standing discrepancies on neurotransmission in non-mammalian and mammalian synapses. The following sections will recapitulate the main findings of a series of studies on sequential steps of synaptic transmission, and present new theoretical framework and conceptual models to account for the experimental data.

## Relationships between the waveform of action potentials and Ca^2+ ^influx

Taschenberger and von Gersdorff (2000) first reported that presynaptic APs at the developing calyx of Held synapse become progressively narrower in half-width but remain unchanged in amplitude during the first two postnatal weeks [19]. Spike narrowing, in this case, arises from speeding of both depolarization and repolarization phases due to up-regulated Na^+ ^and K^+ ^conductances [19,42-45]. To better characterize the impact of spike narrowing on transmitter release, Yang and Wang (2006) took a reductionist approach and employed a series of pseudo-APs to systemically study the distinctive effects of AP depolarization, plateau and repolarization phases on I_Ca _[46]. Slowing depolarization or plateau phase selectively increases I_Ca _by recruiting an increasing number of VGCCs without changing their gating kinetics. Similar changes can also been induced by prolonging the repolarization phase. However, once the I_Ca _amplitude saturates, further broadening serves to slow I_Ca _gating kinetics (both activation and deactivation), which significantly expands the total Ca^2+ ^integral beyond the maximal I_Ca _integral generated by changing AP depolarization or plateau phase. Using paired voltage-clamp recordings of I_Ca _and excitatory postsynaptic currents (EPSCs or I_EPSC_) evoked by pseudo- and real-APs, Yang & Wang (2006) further demonstrated that immature and mature APs (with the half width: 0.4 and 0.27 ms) activate 50% and 35% of the total VGCCs in the terminal respectively, and that the I_Ca_-I_ESPC _relationships are well described by the integral, but not the amplitude, of I_Ca _in the classical Hill function. These observations may help reconcile the opposing views on the effect of spike broadening on presynaptic I_Ca _among different synapses.

At the squid giant synapse, classical work has shown that a single AP opens only ~10% of presynaptic VGCCs [2,47]. In these synapses, AP broadening enhances calcium entry, primarily by increasing the number of activated VGCCs, which manifests as increases in the amplitude of I_Ca _[3]. In contrast, both theoretical simulations and experimental data from central synapses, including the calyx of Held synapse, suggest that an AP effectively opens more than 70% of available VGCCs and that AP broadening increases calcium influx mainly by extending the duration of calcium currents with minimal effect on the amplitude of I_Ca _[8,14-17]. However, these results were obtained largely from immature mammalian central synapses, in which propagating APs usually have wide waveforms (0.5~1 ms) [8,14-17] that typically activate near maximal number of available VGCCs. As a result, AP broadening in these synapses will only reveal changes in the gating kinetics of I_Ca _but not its amplitude. When narrower APs with a half-width of 0.4 ms and 0.27 ms respectively were used as voltage-command templates [46], the fraction of activated VGCCs (50% vs. 35%) was clearly much lower than previously estimated (>70%).

Based on the above, the initial AP width, defined by the depolarization and repolarization time course, likely dictates whether AP broadening induces changes in the amplitude or the kinetics of I_Ca_. It should be noted that experiments in invertebrate synapses are usually conducted at lower temperatures (e.g., <18°C for squid giant synapse) than those in central synapses, which may significantly reduce the fraction of VGCCs recruited by an AP (i.e. 10%) and slow the gating kinetics of these VGCCs. In these aforementioned studies, effects of AP amplitude have not been extensively examined, although Wu et al. suggested that small changes in AP amplitude can profoundly impact quantal output [48]. In the developing calyx of Held synapse where AP amplitude remains stable [46], it appears that speeding of AP depolarization and repolarization rates mainly shortens the AP half-width, reduces the number of VGCCs activated during an AP and accelerates activation and deactivation kinetics of I_Ca_, ultimately ensuring the brevity of Ca^2+ ^transients for synchronized release of transmitters.

## Coupling of Ca^2+ ^influx to vesicular release

Paired recordings from the calyx of Held synapse in both mice and rats reveals a significant leftward shift of the input-output relationship (I_Ca_-I_EPSC_), indicating a developmental upregulation in the release efficiency [46,49]. It has been well documented that the I_Ca _density, or total number of VGCCs on the presynaptic terminal, remains relatively unchanged throughout the development of the calyx of Held synapse [36,50]. This raises the question: how smaller I_Ca_, evoked by a narrow AP at mature synapses, can yield higher quantal output than that evoked by a wide AP at immature synapses? Intuitively, downstream coupling of Ca^2+ ^entry to vesicular release must enhance fusion efficiency to compensate for the reduced presynaptic input.

There are two possible mechanisms underlying such an enhancement: (1) the spatial coupling between VGCCs and SVs in AZs tightens so that the Ca^2+ ^sensors on SVs are exposed to higher local Ca^2+ ^concentrations near the mouth of VGCCs opened during an AP, and/or (2) the Ca^2+ ^sensors (e.g. synaptotagmin) acquire higher Ca^2+ ^sensitivity during development and can readily detect smaller Ca^2+ ^transients from fewer activated VGCCs.

To test the first possibility, Fedchyshyn and Wang (2005) showed that injection of a Ca^2+ ^buffer with slow binding kinetics (EGTA; 10 mM), which can not intercept Ca^2+ ^influx from VGCCs in the immediate vicinity of SVs but is capable of capturing Ca^2+ ^in transit from distant VGCCs, potently attenuated transmitter release in immature terminals (P8-12 mice) as previously demonstrated in rats [26]. Surprisingly, the same manipulation had little effect on mature synapses (P16-18), suggesting that SVs in young synapses are loosely coupled to VGCCs, but transition to a more tightly coupled spatial arrangement during development [36]. Similar conclusion was reached by applying capacitance measurements of exocytosis under different buffer conditions [51]. When a voltage paradigm, which specifically recruited an increasing number of VGCCs without changing kinetics of I_Ca _or the driving force for Ca^2+^, was used to generate a series of graded I_Ca _and I_EPSC_, Fedchyshyn and Wang (2005) estimated Ca^2+ ^channel/domain cooperativity (*m*, the exponent of the I_Ca_-I_EPSC _power relationship), from the slope of linearized Log(I_Ca_)-Log(I_EPSC_) plots (i.e. *I*_EPSC _∝ [*I*_Ca_*]*^*m*^) for immature and mature synapses [36]. Note that *m *derived from this protocol has a distinct meaning from the classical Ca^2+ ^cooperativity (*n*), which is usually interpreted as the number of Ca^2+ ^ions (3~5) which bind cooperatively to the release sensor and trigger vesicular fusion (i.e. molecular cooperativity). The term *m *refers to the mean number of engaged Ca^2+ ^channels/domains (*m*) that cooperatively increase Ca^2+ ^concentration and satisfy the Ca^2+ ^binding requirements of the Ca^2+ ^sensor (*n *= 3~5). *n *is remarkably consistent across experimental systems [49,52] while *m *is more variable [25]. Indeed, it has been shown that *m *values are significantly higher in immature synapses than that in mature synapses, as would be expected from a tighter spatial coupling between VGCCs and SVs with development. This finding implies that the number of VGCCs/Ca^2+ ^domains required for triggering release of single SVs also decreases [36,49,52]. Pharmacological studies with subtype-specific toxins against different VGCCs further demonstrated that transmitter release requires both N- and P/Q-type VGCCs in P8-12 synapses, but becomes entirely dependent on P/Q-type VGCCs in older synapses where these channels are associated with release sites that specifically displayed low *m *values [27,53,54].

Collectively, these observations demonstrate that, at the calyx of Held synapse, there is a developmental transformation from "microdomain," the coupling modality involving cooperative action of many loosely associated N- and P/Q-type VGCCs, to "nanodomain", the coupling modality in which opening of fewer tightly coupled P/Q-type VGCCs effectively gates a fusion event. Incidentally, a recent study by Bucurenciu et al. (2008) showed nanodomain coupling modality also operates at the hippocampal basket cell-granule cell inhibitory synapses [55]. Unlike previous studies debating the coupling nature of VGCCs and SVs between different synapses, these experiments from both excitatory and inhibitory synapses were done in similar conditions ([Ca^2+^]_o_, 1~2 mM), and provided compelling evidence that microdomain and nanodomain coupling modalities are likely distinct physical entities, and may operate in the same or different central synapses depending on their functionalities or developmental stages.

To test the second possibility, two independent studies applied simultaneous Ca^2+ ^imaging and flash photolysis of caged Ca^2+ ^indicators (i.e. DM-nitrophen) with paired patch-clamp recordings to directly measure the sensitivity of the Ca^2+ ^sensors in the developing calyx of Held synapse (P9-11 vs. P16-19 mice, [52]; P8-9 vs. P12-15 rats, [49]). Flash photolysis of caged Ca^2+ ^can produce step-like increases in intracellular Ca^2+ ^concentrations ([Ca^2+^]_i_) in a spatially homogeneous manner in the terminal. Such increases bypass the dynamic coupling process of Ca^2+ ^entry through VGCCs to the release machinery during an AP or a voltage-step, and directly trigger transmitter release correlated with a known [Ca^2+^]_i_. With appropriate experimental paradigms [31,32], the size of readily releasable pool (RRP) and release rates per vesicle at any given [Ca^2+^]_i _in individual synapses are estimated. When release rates are plotted against [Ca^2+^]_i_, their relationship can be fitted with Ca^2+ ^binding-release models similar to the five-site kinetic model proposed by Schneggenburger and Neher (2000). Using this approach, the sensitivity of the release machinery, or the Ca^2+ ^sensor(s), to Ca^2+ ^can be estimated. Surprisingly, experimental data showed that Ca^2+ ^sensitivity of the release sensor in fact decreases with synaptic maturation while molecular cooperativity (*n*) remains unchanged. *K*_D _values increase from ~81 (P9-P11) to ~123 μM (P16-P19) [52]. Similarly, developmental increases in *K*_D _values were also reported in the rat calyx of Held synapse [49]. These studies together led to the conclusion that changes in the spatial coupling between VGCCs and SVs, but not the sensor sensitivity, accounts for developmental upregulation of quantal output at the calyx of Held synapse.

## Mechanisms underpinning developmental plasticity in AP-evoked transmitter release

When all aforementioned experimental results are taken into consideration, we can readily resolve the issue of how decreasing I_Ca_, due to AP narrowing in the developing calyx of Held synapse, becomes more effective in triggering transmitter release. The answer resides in the two compensatory developmental processes in which AP narrowing attenuates the total Ca^2+ ^influx during an AP, while increasingly intimate spatial coupling of VGCCs to SVs in AZs enhances fusion efficiency [36,46,49,50,52]. These opposing effects are, at the very least, independent of changes in the sensitivity of the Ca^2+ ^sensors on SVs but most likely also serve to compensate for decreasing sensitivity with development [49,52]. From a physiological perspective, both developmental AP narrowing and transformation of the release modality are important adaptations allowing the calyx of Held synapse to function as a temporally precise relay in the auditory brainstem. Narrow APs enable high-frequency firing by shortening the refractory period of APs while brief Ca^2+ ^transients during APs lead to the rapid rise and collapse of local [Ca^2+^] nanodomains, promoting highly synchronized release with minimal synaptic delay, temporal jitter, and asynchronous release during transmission at mature calyx synapses. Short Ca^2+ ^transients will also reduce the likelihood of presynaptic Ca^2+ ^overload that can occur during prolonged high-frequency transmission, lowering the energy required for Ca^2+ ^clearance via ion pumps and exchangers [56]. Therefore, the combination of AP narrowing and concurrent tightening of spatial coupling between VGCCs and SVs are critical developmental adaptations enabling the calyx of Held synapse to control the quantal output per AP and the efficient use of the RRP of SVs during high-frequency neurotransmission.

## Conceptual models for topographic organization of SVs and VGCCs at the release site

With accumulating evidence from the developing calyx of Held synapse, it is important to further conceptualize the spatial reorganization of VGCCs and SVs in AZs to interpret experimental data. However, lack of precise knowledge of the subsynaptic distribution patterns of VGCCs, their single-channel conductance at physiological concentrations of [Ca^2+^]_o_, and the number of VGCCs per AZ, prevents establishment of a realistic model for release site topography. Nevertheless, measurements of the Ca^2+ ^channel/domain cooperativity (*m*) and the EGTA induced attenuation of transmitter release provide sufficient constraints for establishing theoretical models to recapitulate the nature of Ca^2+^-secretion coupling at the developing calyx of Held synapse.

**Figure 1:**
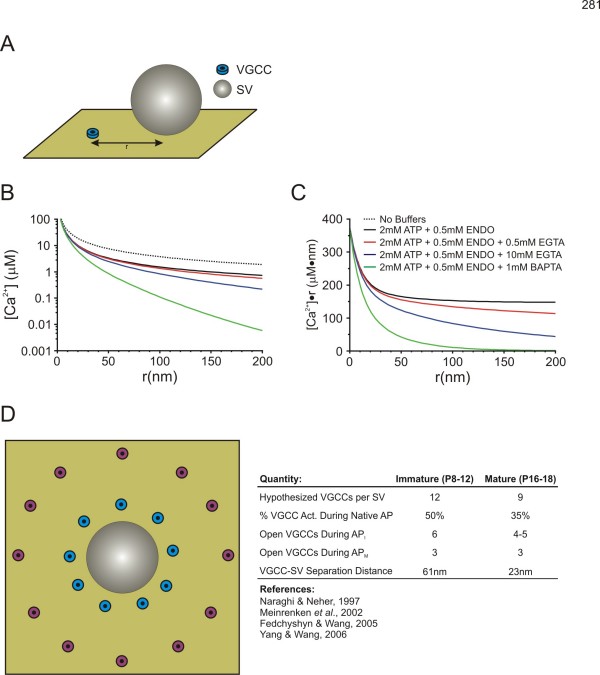
**Linearized Buffered Diffusion & Release Site Topography Model**. (A) Schematic representation of the single VGCC for which the buffered diffusion model determines [Ca^2+^] as a function of r. (B) [Ca2+] as a function of r for the various buffer conditions from Fedchyshyn & Wang (2005) in response to the steady-state opening of a single VGCC. (C) [Ca2+]·r as a function of r, or the buffer composition "fingerprint" for the various buffer conditions from Fedchyshyn & Wang (2005) in response to the steady-state opening of a single VGCC. (D) Modeled arrangement of VGCCs in immature (magenta VGCCs) and mature (cyan VGCCs) synapses. ENDO refers to endogenous immobile buffer. Single VGCC flux for all panels F = 6.24 × 105 ions/sec or 0.2pA of constant current. Buffer parameters are as in Naraghi & Neher (1997). Schematics are not drawn to scale.

The linearized buffered Ca^2+ ^diffusion model of Naraghi and Neher (1997) allows for the calculation of the [Ca^2+^] at any distance from the mouth of a single VGCC (Figure 1, also see Appendix 1 for details) provided that the composition of Ca^2+ ^buffers contained within a presynaptic recording pipette is known [57]. A particularly useful aspect of a linearized system is that it allows for the use of the superposition principle in considering greater numbers of VGCCs. Thus, the peak [Ca^2+^] can be determined at an arbitrary distance (**r**) from any desired arrangement of VGCCs simply by summating their individual contributions. This freedom allows us to assume any distribution of VGCCs around a SV while being able to determine the [Ca^2+^] at that point. This provides a constraint governing the peak [Ca^2+^] experienced by the release sensor for a particular set of buffer conditions.

To determine the [Ca^2+^] at the release sensor, we selected a simple ring of VGCCs, at equal **r **from the SV, as a simplified topography. It is well established that, at least in the immature synapse, VGCCs are most likely at variable distances from their SV, located in clusters or some other heterogeneous spacing arrangement [58-60]. However, assuming a more simplistic arrangement of a ring of VGCCs simplifies the determination of [Ca^2+^] arising from multiple VGCCs and sufficiently address whether VGCC-SV coupling differences can account for developmental changes in SV release at this synapse.

Previous modeling studies, performed with data acquired from the immature synapse, have hypothesized an average VGCC-SV separation of ~80 nm [58]. In addition, morphological data, at least from the immature synapse, can provide some constraints when choosing and testing potential VGCC-SV separation distances. AZs at the immature calyx of Held are approximately 0.01 μm^2 ^in area [61]. If we assume that the AZ is approximately circular in shape, then the diameter of the AZ is ~120 nm and allows for two docked SVs on average [50,60]. This approximate AZ geometry implies that VGCCs can be separated by no more than ~100 nm from any given SV, assuming VGCCs are in the AZ, thus providing an upper limit on the testing ranges for VGCC-SV separation. Similarly, the minimum separation distance between SVs and VGCCs is limited by the presence of the release machinery, and its many associated proteins, at the base of the SV. The SNARE complex (ring) is estimated to have a diameter of ~20 nm or larger depending on the size of the SV [61], thus we considered 20 nm to be a lower limit for VGCC-SV separation in our modeled SV arrangements.

Given these limits, we simulated a number of possible VGCC-SV separation distances with different numbers of VGCCs contributing to the [Ca^2+^] transient in each case (see Appendix 1 and Table 1 for parameters). Upon invasion of an immature AP into the calyx of Held, approximately 50% of VGCCs open [46] and the Ca^2+^-domain cooperativity values from immature synapses is 5~6 [36]. Therefore, we summated the [Ca^2+^] contribution from 6 of the 12 VGCCs and set this as the peak [Ca^2+^] for the input into a kinetic release model [31,32,57,62]. For the immature synapse, we found that a ring of 12 VGCCs at a distance of **r **= 61 nm reproduce experimental data well, in line with the average spacing determined by Meinrenken *et al*. (2002) [58]. Given that VGCC-SV coupling tightens in the mature synapse and fewer VGCCs are required to release a SV, we hypothesized a different topography of VGCCs from that of the immature synapse. In the mature synapse where the narrower APs activate approximately 35% of VGCCs [46] and Ca^2+ ^channel/domain cooperativity values for this developmental stage is around 3 [36], we found that the summated contribution of 3 channels per AP from a ring of 9 VGCCs at a mean distance of **r **= 23 nm to the peak [Ca^2+^] reproduces our experimental findings well. These models represent a simplified interpretation of release site topography which allows for simplification of the calculations required to reproduce main experimental observations including developmental increases in quantal output and the extent of EGTA-induced attenuation of EPSCs in immature and mature synapses.

**Table 1:** Parameters for Buffered Ca^2+ ^Diffusion Models

Quantity	Immature (P8-12)	Mature (P16-18)	Reference
ATP Diffusion Constant	220 μm^2^/s	220 μm^2^/s	57
K_d _(ATP)	2300 μM	2300 μM	57
k_on _(ATP)	500 μM^-1^•s^-1^	500 μM^-1^•s^-1^	57
[ATP]_pipette_	2000 μM	2000 μM	57

EGTA Diffusion Constant	220 μm^2^/s	220 μm^2^/s	57
K_d _(EGTA)	0.18 μM	0.18 μM	57
k_on _(EGTA)	2.5 μM^-1^•s^-1^	2.5 μM^-1^•s^-1^	57
[EGTA]_pipette_	500/10000 μM	500 μM	36

BAPTA Diffusion Constant	220 μm^2^/s	220 μm^2^/s	57
K_d _(BAPTA)	0.22 μM	0.22 μM	57
k_on _(BAPTA)	400 μM^-1^•s^-1^	400 μM^-1^•s^-1^	57
[BAPTA]_pipette_	1000 μM	1000 μM	36

ENDO Diffusion Constant	15 μm^2^/s	15 μm^2^/s	57
K_d _(ENDO)	50 μM	50 μM	57
k_on _(ENDO)	100 μM^-1^•s^-1^	100 μM^-1^•s^-1^	57
[ENDO]_pipette_	500 μM	500 μM	57

Ca2+ Diffusion Constant	220 μm^2^/s	220 μm^2^/s	57
Resting [Ca^2+^]_i_	50 nM	50 nM	58
Steady-State Single VGCC Flux	6.24 × 10^5 ^ions/s	6.24 × 10^5 ^ions/s	24

## Spatiotemporal profile of Ca^2+ ^transients seen by Ca^2+ ^sensors on SVs during an AP

**Figure 2:**
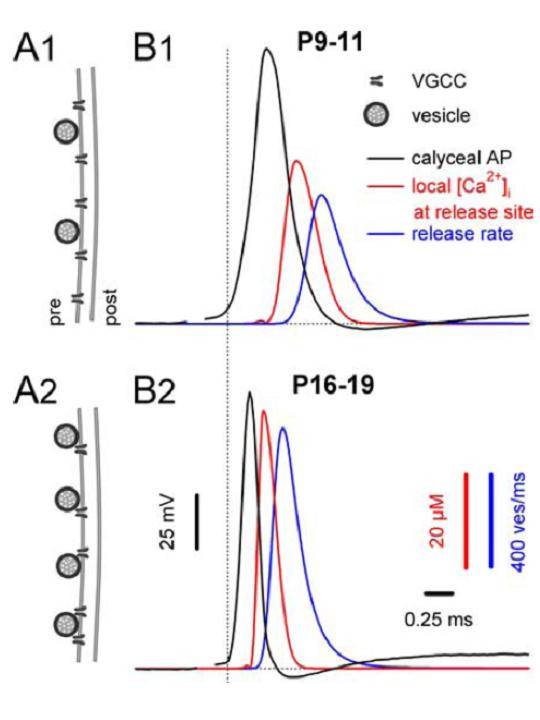
**Summary of developmental changes in the temporal profiles of the local [Ca^2+^]_i _transient seen by the Ca^2+ ^sensor and glutamate release during an AP**. **A**, Schematic diagram illustrating developmental tightening in the coupling between synaptic vesicles and VGCCs. Immature calyces (**A1**) have fewer docked vesicles that are loosely coupled to VGCCs, whereas mature calyces (**A2**) possess approximately a twofold larger number of release-competent vesicles that more closely colocalize with Ca^2+ ^channels. **B**, Timing (right) of the local [Ca^2+^]_i _(red) and release (blue) transients relative to the presynaptic APs (black) for P9--P11 (**B1**) and P16--P19 (**B2**) calyces. Traces in **B1 **and **B2 **were aligned relative to the onset of the presynaptic APs (dotted line). The average time course of the AP-evoked local [Ca^2+^]_i _transients was obtained from 16 of P9--P11 and 18 of P16--P19 synapses. The peak of the local [Ca^2+^]_i _transient occurred ~410 μs earlier in mature synapses, because of their faster and briefer presynaptic APs (Image © Wang et al., 2008).

The rise and collapse of local Ca^2+ ^domains near the mouth of VGCCs during APs are difficult to directly resolve (but see [62]). However, these can be quantitatively extrapolated from the spatiotemporal profiles of local Ca^2+ ^by using parameters obtained from kinetic models describing the [Ca^2+^]_i_-release rate curves derived from flash photolysis experiments [32]. Ca^2+ ^transients are reproduced by first deconvolving the release profiles of AP-evoked EPSCs, using the respective average mEPSC waveforms, followed by simulating the local [Ca^2+^]_i _transients at the sites of vesicle fusion required to match the release profiles of individual synapses. This can be achieved by using inverted waveforms of the simulated calyceal I_Ca_, beginning with the Hodgkin-Huxley (HH) model [16] as the initial approximation, and then gradually adjusting the amplitude and decay time courses of [Ca^2+^]_i _transients until the experimental release profiles of native synapses are accurately reproduced. Simulations performed from data recorded in the mouse calyx of Held, as summarized in Figure 2, demonstrated that with developmental tightening of the spatial coupling between VGCCs and SVs, the local [Ca^2+^]_i _"seen" by the Ca^2+ ^sensors increases from 35 to 56 μM and the SV release rate increases from ~600 SVs/ms to 1000 SVs/ms despite smaller and briefer I_Ca _[52]. Similar results, regarding developmental increased in peak [Ca^2+^]_i_, were also derived from the developing rat calyx of Held synapse [49].

These results reinforce the idea that tightening of the spatial coupling between VGCCs and SVs plays a predominant role over other developmental adaptations in enhancing quantal output at the calyx of Held synapse. This observation may also explain the significant discrepancies in estimated AP-driven peak [Ca^2+^]_i _values (10~300 μM) described in previous work [28-33]. That is, this heterogeneity in the distance between VGCCs and SVs in different experimental systems may contribute to observed variations in the measurement of *Pr *values in this and other central synapses.

## Concluding remarks & prospects

The calyx of Held synapse has emerged as one of the most prominent and important preparations in the field of synaptic transmission and developmental plasticity. Studies performed in this model have addressed many important and previously unresolved issues related to the fraction of VGCCs evoked by APs, changes in the amplitude and kinetics of I_Ca _during spike broadening, the downstream coupling modalities between VGCCs and SVs, as well as the efficacy of Ca^2+^-dependent transmitter release. In particular, experimental data and theoretical simulations that confirm the developmental transformation from microdomain to nanodomain release modalities is of central importance for reconciling opposing views on the nature of coupling between VGCCs and SVs and for presenting the proof-of-principle for idealistic topographic arrangements of VGCCs and SVs in AZs.

However, these studies have also raised new questions. Since a majority of functional and morphological adaptations at the developing calyx of Held synapse appear to occur after the onset of hearing (at P11/12), the question remains, what are the roles of sensory activity, if any, in driving developmental plasticity and what signaling cascade(s) are involved in this transformation[63]. And, is functional plasticity reciprocally coupled to morphological remodeling at gross and subsynaptic levels during development? Furthermore, neither the molecular substrates differentiating microdomain and nanodomain coupling modalities, nor the exact topographic arrangements for these distinct release modalities, are known. Many synaptic proteins including syntaxin, Rim, Munc, CAST, synaptotagmin and etc have been implicated in interactions with VGCCs and "positional priming" (i.e. a process for SVs to become tightly associated with Ca^2+ ^influx) in the vicinity of release sites before fusion [64], but critical elements underpinning the proximity of VGCCs and SVs remain mysterious. With the increasing availability of transgenic mice, in which key synaptic proteins are deleted or mutated, as well as potential applications of novel viral infection and sRNAi technologies *in vivo *[65], future work at the calyx of Held synapse will undoubtedly lead to further ground breaking discoveries to resolve these critical questions. As answers come to the forefront from this unique synapse, it will undoubtedly continue to make indispensable contributions to our understanding of this and other central synapses.

## Competing interests

The authors declare that they have no competing interests.

## Authors' contributions

LYW, MJF and YMY wrote this article jointly. LYW designed the conceptual model and MJF performed computer simulations. All authors read and approved the final manuscript.

## Appendix 1

If the standing [Ca^2+^] gradients are assumed to be at steady-state around an open VGCC and buffer saturation is small, the differential equations describing buffered Ca^2+ ^diffusion can be linearized, considerably simplifying the mathematical description of the system [57,66]. The assumption of low buffer saturation has been proven valid, even for Ca^2+ ^buffers with high binding ratios and short length constants like BAPTA and conservatively assuming single VGCC flux of around 0.3 pA or smaller [57]. In our simulations, this approximation should also hold as we have chosen single VGCC flux to be well below this threshold at 0.2 pA.

At steady state, the [Ca^2+^] carried by three Ca^2+ ^buffers can be described by the following;(1)

Where Φ is the single VGCC flux and;

D_N _is the diffusion coefficient of the N^th ^buffer species and;

In these equations, B_N _is the concentration of the N^th ^buffer species, K_N _is the dissociation constant of the N^th ^buffer species, k_Noff _is the Ca^2+ ^unbinding rate of the N^th ^buffer species, and k_Non _is the Ca^2+ ^binding rate of the N^th ^buffer species. The choices for the above parameters are shown in Table 1 and are taken directly from Naraghi & Neher (1997) with the exception of Φ which was taken as an average from Stanley (1993) and Shahrezaei & Delaney (2004). Given the necessary parameters, equation 2 can be solved to determine the [Ca^2+^] at any distance **r **from a single VGCC for N described buffer species.

Using the above equations, we have shown the [Ca^2+^] as a function of **r **for various buffer compositions (Figure 1B). In addition, Figure 1C shows [Ca^2+^]•**r **as a function of **r**, which eliminates the 1/**r **dependence of [Ca^2+^] diffusion, and shows more clearly the characteristic length constant of the given buffer compositions (as in Naraghi & Neher, 1997). Note that the hypothesized endogenous mobile buffer properties, recently uncovered at the immature calyx of Held, have not been included here [67]. This assumption is due to the fact that any endogenous mobile buffers contained within the calyx of Held are likely dialyzed rapidly with those contained in the much larger volume of the presynaptic patch pipette.
